# Nanostructured Cobalt Doped Barium Strontium Titanate Thin Films with Potential in CO_2_ Detection

**DOI:** 10.3390/ma13214797

**Published:** 2020-10-27

**Authors:** Cristina F. Ciobota, Roxana M. Piticescu, Ciprian Neagoe, Ioan A. Tudor, Alexandru Matei, Dumitru V. Dragut, Arcadie Sobetkii, Elena M. Anghel, Adelina Stanoiu, Cristian E. Simion, Ovidiu G. Florea, Simona E. Bejan

**Affiliations:** 1 Laboratory of Advanced and Nanostructured Materials, National Research & Development Institute for Non-Ferrous and Rare Metals—IMNR, 102 Biruintei Blvd., 077145 Pantelimon, Romania; rusticristina@yahoo.com (C.F.C.); roxana.piticescu@imnr.ro (R.M.P.); ciprian.neagoe@imnr.ro (C.N.); atudor@imnr.ro (I.A.T.); alex.matei@imnr.ro (A.M.); dragutv@imnr.ro (D.V.D.); 2 MGM Star Construct SRL, 7 Pancota Street, Building 13, 022773 Bucharest, Romania; office@mgmstar.ro; 3 Oxide Compounds and Materials Science Laboratory, “Ilie Murgulescu” Institute of Physical Chemistry of the Romanian Academy, 202 Splaiul Independentei, 060021 Bucharest, Romania; manghel@icf.ro; 4 Laboratory of Atomic Structures and Defects in Advanced Materials, National Institute of Materials Physics, Atomiştilor 405A, P.O. Box MG-7, 077125 Măgurele, Romania; adelina.stanoiu@infim.ro (A.S.); simion@infim.ro (C.E.S.); ovidiu.florea@infim.ro (O.G.F.)

**Keywords:** hydrothermal synthesis, Co-doped BaSrTiO_3_ perovskite nanopowders, RF sputtering method, Co-doped BaSrTiO_3_ thin film, CO_2_ detection

## Abstract

In this work, (Ba_0.75_Sr_0.25_) (Ti_0.95_Co_0.05_) O_3_ perovskite nanostructured material, denoted subsequently as Co-doped BaSrTiO_3_, was synthesized in a one-step process in hydrothermal conditions. The obtained powder was heat-treated at 800 °C and 1000 °C, respectively, in order to study nanostructured powder behavior during thermal treatment. The Co-doped BaSrTiO_3_ powder was pressed into pellets of 5.08 cm (2 inches) then used for thin film deposition onto commercial Al_2_O_3_ substrates by RF sputtering method. The microstructural, thermal, and gas sensing properties were investigated. The electrical and thermodynamic characterization allowed the evaluation of thermodynamic stability and the correlation of structural features with the sensing properties revealed under real operating conditions. The sensing behavior with respect to the temperature range between 23 and 400 °C, for a fixed CO_2_ concentration of 3000 ppm, highlighted specific differences between Co-doped BaSrTiO_3_ treated at 800 °C compared to that treated at 1000 °C. The influence of the relative humidity level on the CO_2_ concentrations and the other potential interfering gases was also analyzed. Two possible mechanisms for CO_2_ interaction were then proposed. The simple and low-cost technology, together with the high sensitivity when operating at room temperature corresponding to low power consumption, suggests that Co-doped BaSrTiO_3_ has a good potential for use in developing portable CO_2_ detectors.

## 1. Introduction

Recent progress in manufacturing advanced materials and technologies has seen the development of novel sensorial thin films for the rapid detection of a wide range of environmentally-polluting industrial waste. Industrial gases containing CO, NO_x_, NH_3_, CH_4_, SO_2_, and CO_2_, as well as exhaust gases from automobiles, require special detection and monitoring sensors to avoid harm to humans and the surrounding atmosphere [1]. The notable increase in public health issues and climate threats requires huge efforts in order to reduce the impact of these dangerous gases. The development of efficient monitoring and control of low-cost gas sensors could be the first step to address such a challenge.

Barium strontium titanate (BST, Ba_x_Sr_1−x_TiO_3_) is a material with a perovskite structure that has attracted the interest of researchers and engineers due to its ferroelectric properties and composition correlated to Curie temperature [2]. BST has been used successfully in high voltage capacitors, tunable filters, detectors, piezoelectrics, sensors, pressure transducers, actuators, and optoelectronic devices [3,4,5,6,7].

Controlling the amount of n-type (donor) and p-type (acceptor) dopants of pure barium titanate (BT) materials can modify semiconductor behavior. In order to improve the electrical properties of pure barium titanate, different types of donor or acceptor dopants have been employed. Small amounts of donor dopant at the A (Ba^2+^) site (La^3+^, Ce^3+^, Dy^3+^, Nd^3+^, Gd^3+^, Sm^3+^, Y^3+^) or acceptor dopant at the B (Ti^4+^) site (Zr^4+^, Sn^4+^, Nb^5+^, Sb^5+^, Ta^5+^) in a barium titanate structure have been investigated for a better understanding of semiconducting behavior (minimum resistivity occurrence is at room temperature for dopant at Ba^2+^ or Ti^4+^ positions) [8]. The electronic properties of Ba_x_Sr_1−x_TiO_3_ can be modified when Sr^2+^ partially substitutes Ba^2+^ in pure BaTiO_3_. Besides the doping process, the electrical properties of BT are affected by microstructure, grain size, and compositional design [9].

Barium strontium titanate (Ba_x_Sr_1−x_TiO_3_) is a versatile material that is studied intensively nowadays for a variety of sensor applications, such as piezoelectric, pyroelectric, humidity, and gas sensing [10,11,12,13]. BST nanostructures have been synthesized by various methods or processes, such as sol-gel [14], co-precipitation [15], solvothermal [16], reverse microemulsion [17], pulsed laser deposition [18], ball milling [19], and hydrothermal techniques [20,21].

Hydrothermal technology has the advantage of producing powders with a high degree of crystallinity, fine particle size, and narrow particle size distribution. By varying the synthesis parameters, such as reaction temperature, time, and pH values, one can control the synthesis process. The available precursors for BaSrTiO_3_ preparations in hydrothermal conditions are inexpensive and easy to handle. For this reason, the hydrothermal synthesis of BaSrTiO_3_ is the best option taking into account the economic and technological aspects [22].

In our study, the doping mechanism is achieved by partial substitution of Ba^2+^ ions (A site) by Sr^2+^ and Ti^4+^ ions (B site) by Co^3+^ in the ABO_3_ structure using hydrothermal processes [10]. Thus, an excess of free charge carriers is created. This paper aims to investigate the microstructural, thermal, and gas sensing properties of nanostructured (Ba_0.75_Sr_0.25_) (Ti_0.95_Co_0.05_) O_3_ perovskite powder (Co-doped BaSrTiO_3_) synthesized by the hydrothermal method. Electrical and thermodynamic characterization (by Differential Scanning Calorimetry (DSC) for mass loss and transient plane source for thermal conductivity) allows the evaluation of thermodynamic stability and the correlation of structural features with electrical conductivity under gas conditions. Two possible mechanisms of carbon dioxide (CO_2_) detection are then proposed under real operating conditions (i.e., in the presence of 50% relative humidity (RH)).

Carbon dioxide (CO_2_) is a colorless, inert gas. Along with other natural gases, it is a greenhouse gas, which is likely to occur either naturally (via organic matter decomposition) or from human activities (such as fossil fuel burning for power generation, oil refining, natural gas production, and/or transportation). When the CO_2_ concentration is higher in the surrounding atmosphere, a direct impact is likely to occur through global climate changes. The maximum limit of CO_2_ required by safety regulations within a gaseous mixture of other potential interfering agents found in mines must be between 5 to 20%. Beyond this limit, CO_2_ becomes hazardous for working personnel. Among the already mentioned issues, one should keep in mind that the direct impact of CO_2_ release in the atmosphere is leading to global climate changes, such as the greenhouse effect and global warming [23]. Therefore, one of the major scientific concerns in the gas-sensing field is linked to the possibility that the qualitative and quantitative detection of CO_2_ is of substantial interest in various fields by using gas sensors capable of detecting low traces under different working conditions [24]. State of the art CO_2_ detection comes at a high price, or with complicated operation mode sensors [25,26,27] or low selective chemo-resistive sensors operating at high temperatures [28,29]. In the present paper, we report a simple, sensitive, and room temperature operating chemo-resistive CO_2_ sensor, based on Co-doped BaSrTiO_3_ obtained by a simple and low-cost technology.

## 2. Materials and Methods

### 2.1. Co-Doped BaSrTiO_3_ Hydrothermal Synthesis

**Figure 1:**
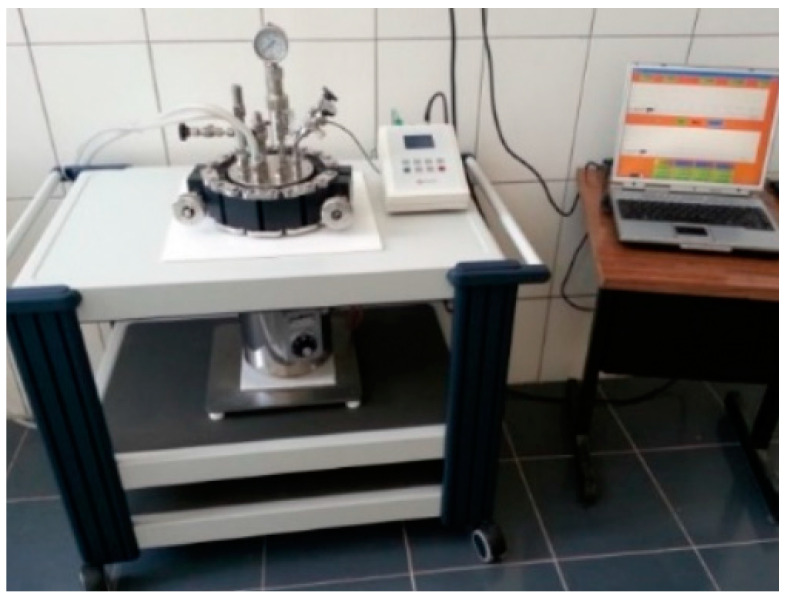
The Berghof autoclave experimental device.

Nanostructured perovskite material (Ba_0.75_Sr_0.25_) (Ti_0.95_Co_0.05_) O_3_ encoded as BSTCo5 was synthesized in a one-step process by hydrothermal method starting from water-soluble salts of Ti, Ba, Sr, and Co, respectively. TiCl_4_ (Sigma-Aldrich, St. Louis, MI, USA, p.a. 98%), Sr (NO_3_)_2_ (Merck, Kenilworth, NJ, USA, p.a. >99%), and Ba (OH)_2_ 8H_2_O (Merck, e p.a. >98%) were used as precursors, and KOH was used as a mineralizer. The amounts of Ba (OH)_2_ 8H_2_O, Sr (NO_3_)_2_, Co (NO_3_)_2_ 6H_2_O (Sigma-Aldrich, p.a. >99%), and TiCl_4_ were established in agreement with the theoretical molar formula (Ba_0.75_Sr_0.25_) (Ti_0.95_Co_0.05_) O_3_. The precipitation reaction pH was in the range of 9.5–13. The suspension thus obtained was subjected to hydrothermal processing (pressure 40 atm, temperature 200 °C, and time 2 h). The final suspension was introduced into a Teflon vessel of a sealed hydrothermal autoclave reactor (5 L, Berghof Products + Instruments GmbH, Berghof, Germany (Figure 1)) endowed with a cooling system. To control the reaction pressure, argon gas was purged inside the autoclave, according to working procedure described in [30].

The Teflon vessel was filled to 80% of its capacity. To control the size and shape of the particles, after hydrothermal treatment, the autoclave was cooled at 2 °C/min until it reached 50 °C.

The wet precipitate was then filtered, washed with distilled water, and dried in an oven at 100 °C for 8 h, followed by hydrothermal synthesis. In order to study nanostructured powder behavior during thermal treatment, samples of the as-obtained BSTCo5 were subjected to heat treatment at either 800 °C and 1000 °C for two hours, and the samples were encoded as BSTCo5_800 and BSTCo5_1000, respectively. The optimum parameters for powder processing of RF-sputtering targets were selected after a close analysis of all the thermodynamic, morphological, and structural data.

### 2.2. Deposition Method of Sensing Layer

**Figure 2:**
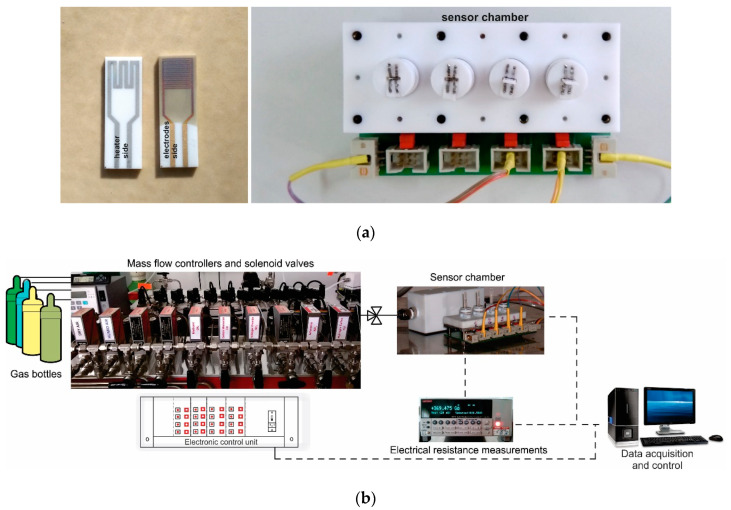
(**a**) Details about electrical connections of the sensor chamber and sensor layout; (**b**) Gas mixing system workbench with data acquisition.

The Co-doped BaSrTiO_3_ perovskite powder obtained as described above, was processed and pellets of 5.08 cm (2 inches) were obtained under 5 tons/cm^2^ force. In order to be used as targets in the RF sputtering process, the pellets were then sintered for one hour at 1000 °C. The RF sputtering system consisted of a TORUS 2HV magnetron source, KJLC 300 W Kurt J. Lesker power supply, VUP-5 vacuum installation, mounting and rotating supports, and a cathode cooling system. Thin films were deposited under 75 W sputtering power, 150 VDC, and 10 Pa argon vacuum conditions onto commercial Al_2_O_3_ substrates (Metrohm DropSens, Oviedo, Spain) provided with Au interdigital electrodes for measuring the sensitive layer resistance and Pt heaters for holding the sensor at the desired operating temperature (Figure 2a). The thin films were deposited under a controlled speed over 3 h and further heat-treated for 20 min at 800 °C under vacuum conditions in order to improve film adherence and crystallinity (BI no. 131119/30.07.2019) [31].

### 2.3. Characterization

Powders were characterized from the chemical point of view by the Inductively Coupled Plasma Atomic Emission Spectroscopy (ICP-OES) technique, using ICP-OES 725 (AGILENT, Santa Clara, California, SUA) equipment, according to ASTME E 1479-16 standards.

The microstructure of the powders was examined using a Bruker D8 ADVANCE (Bruker AXS GmbH, Karlsruhe, Germany) X-ray Diffractometer with Bragg–Brentano geometry using Cu-Kα characteristic radiation and equipped with a scintillation counter and a graphite monochromator. The data was collected at a step size of 6 s per step and a collection angle 2θ = 20°–80°, at room temperature. The data acquisition and the identification of the phases contained within the samples was performed using the software package DIFFRAC SUITE.EVA (5.1 Bruker AXS GmbH, Karlsruhe, Germany) and the ICDD PDF-4+ 2020 database, edited by the International Center for Diffraction Data (ICDD).

The UV Raman spectra were collected by means of a LabRAM HR800 (HORIBA FRANCE SAS, Palaiseau, France) spectrometer equipped with a CCD HeCd laser (325 nm) from Kimmon Koha (Tokio, Japan) and a 2400 grating. A microscope objective with magnification of 40× NUV (NA 0.47) from Olympus (Tokyo, Japan) was used to focus the laser light on the samples.

Scanning Electron Microscopy was performed using a FEI Quanta 250 (FEI Company, Eindhoven, Netherlands) high resolution microscope incorporated with an Energy Dispersive X-Ray Spectrometer, produced by EDAX Ametek (Mahwah, NJ, USA), consisting of an Element Silicon Drift Detector and a Team 4.5 EDS Analysis System (EDAX Ametek, Mahwah, NJ, USA). The samples were prior coated with a thin gold layer in order to improve the conduction of the sample.

Size distribution of the material was measured by DLS instrument (Zetasizer Nano ZS90; Malvern Instruments, Malvern, UK) using a 4 mW He-Ne laser at a wavelength of 632.8 nm. Briefly, 0.05 g of the sample was mixed with deionized water, ethanol, and poly(acrylic acid sodium salt) solution from Sigma-Aldrich (St. Louis, MI, USA) in a predetermined proportion.

The thermal behavior of Co-doped BaSrTiO_3_ samples under nonisothermal conditions was investigated by Differential Scanning Calorimetry (DSC) and thermogravimetry (TG) using SETSYS Evolution 17 (Setaram, Caluire-et-Cuire, France) equipment. The heat flow was calibrated and adjusted at different temperatures using certified materials at 3 heating rates. The samples were heated in alumina crucibles, in a temperature range from 25 to 1200 °C, under a constant flow of pure argon (16 mL/min), and a 10 K min^−1^ heating rate.

Thermal conductivity was measured at room temperature by transient plane source using the hot disk method (TPS 2200, Hot Disk, Göteborg, Sweden) on pairs of cylindrical samples (11 mm diameter and 6 mm height) heat-treated at 800 °C. Briefly, a 2 mm diameter hot disk Kapton sensor (code 7577) sandwiched between the two cylindrical replicate samples was used to simultaneously generate heat and monitor the temperature changes. The method directly measured the thermal conductivity, using a calculation algorithm implemented by the manufacturer as the instrument data processing software.

Gas sensing properties of previously obtained sensors were placed into the sensor chamber (Figure 2a) and evaluated under real operating conditions (the presence of relative humidity and other potential interfering gases) simulated in the laboratory using a dynamic Gas Mixing System (GMS) [32]. The total flow through the system consisted of mixed user-defined gas types and could be adjusted up to 200 mL/min. The CO_2_ gas concentrations could be adjusted from 400 to 3000 ppm, whereas other potential interfering gases at their specific detection limit ppm levels (CO, CH_4_, NO_2_, H_2_S, NH_3_, and SO_2_) were dosed in synthetic air (purity 5.0) with variable relative humidity (0–50% RH). Real-time data acquisition of the electrical resistance changes was acquired using a Keithley Electrometer 6517A (Cleveland, Ohio, USA) (see Figure 2b).

## 3. Results and Discussion

Chemical, morphological, structural, thermal, and conductivity analyses were performed to better understand the behavior of the nanostructured powder during processing and deposition by RF sputtering. The results are presented below.

### 3.1. Chemical Composition of Co-Doped BaSrTiO_3_ Powder

Chemical composition was determined for Co-doped BaSrTiO_3_ as obtained by hydrothermal procedure. Ba, Sr, Ti, Co were determined by ICP-OES and CO_3_^2−^ was determined by gravimetric analysis. CO_3_^2−^ analysis (0.8 ± 1%) indicated a 96% powder purity.

Spectroscopy was carried out according to ASTM E1479-16. The results of elemental analysis are presented in Table 1.

### 3.2. X-ray Diffraction of Co-Doped BaSrTiO_3_ Powder

**Figure 3:**
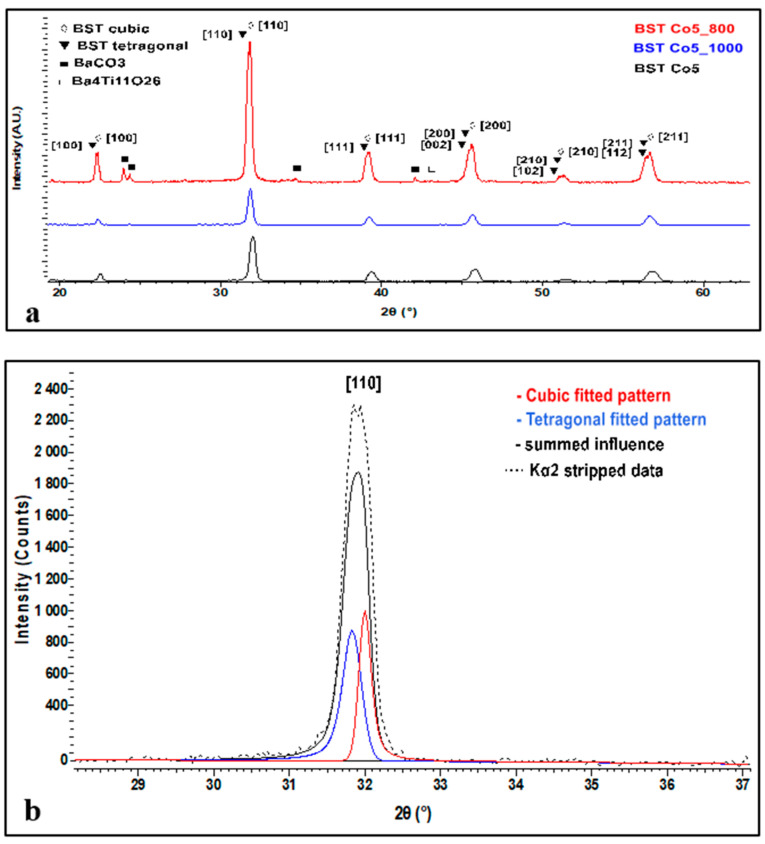
XRD analysis of BSTCo5, BSTCo5 _800, and BSTCo5_1000 (**a**), details of the cubic-tetragonal structure (**b**).

Figure 3 illustrates patterns of BSTCo5, BSTCo5_800, and BSTCo5_1000.

The main reflections at 2θ around 22°, 31°, 38°, and 45° can be indexed with both tetragonal (P4mm PDF 70-9164) and cubic (Pm-3m PDF 70-9165) polymorphs of the perovskite material. Since the intensity of the main diffraction peak of the BaCO_3_ phase is quite weak, this indicates traces of this phase in the sample.

The XRD spectrum in Figure 3a presents all diffraction peaks related to the BST phase. In general, the sample related contribution to the diffraction peak profiles is often described as a combination of Gauss and Lorentz (Cauchy) functions. When viewing the profile of the peaks, it is clear that they are affected by the influence of two or more phases. Thus, in Figure 3b, we present the fitted patterns of both cubic and tetragonal peaks related to the [110] crystallized plane, which precisely explains the peak profile and shape. This clearly indicates the presence of both structures.

For Co-doped BaSrTiO_3_, on the [110] crystallization direction, the crystallite size was calculated by taking into consideration a split pseudo-Voigt type of function and using Scherrer equation with consideration to the FWHM. The calculated crystallite size was around 43.5 nm for the cubic structure and around 27.8 nm for the tetragonal structure.

One can observe in the sample heat-treated at 1000 °C (encode BSTCo5_1000) that a small amount of secondary phase Ba_4_Ti_11_O_26_ (PDF 83-1459) appears (low-intensity diffraction lines at 2θ angles 27 and 30°).

In literature, this phase appears in Fe_2_O_3_ doped BaTiO_3_ powder [33]. This phase was also identified in a (Ba_0.2_Sr_0.2_Ca_0.2_Mg_0.2_Pb_0.2_) TiO_3_ perovskite compound when it was heat-treated at 1400 °C [34] in an Sb-doped barium strontium titanate powder (with TiO_2_ and SiO_2_ excess), which was heat-treated at 1000 °C [35]. If we take into account the aforementioned studies, it seems to be a high-temperature related phase.

XRD investigation completes the result given by the elemental chemical composition analysis. The target compound was not subjected to XRD analysis as previous experience indicated that the presence of BaCO_3_ in BST heat-treated samples diminishes almost entirely [13].

The XRD pattern of the BST sample obtained by the hydrothermal method is presented in Figure 3.

### 3.3. UV Raman Spectroscopy for Co-Doped BaSrTiO_3_ Powder

**Figure 4:**
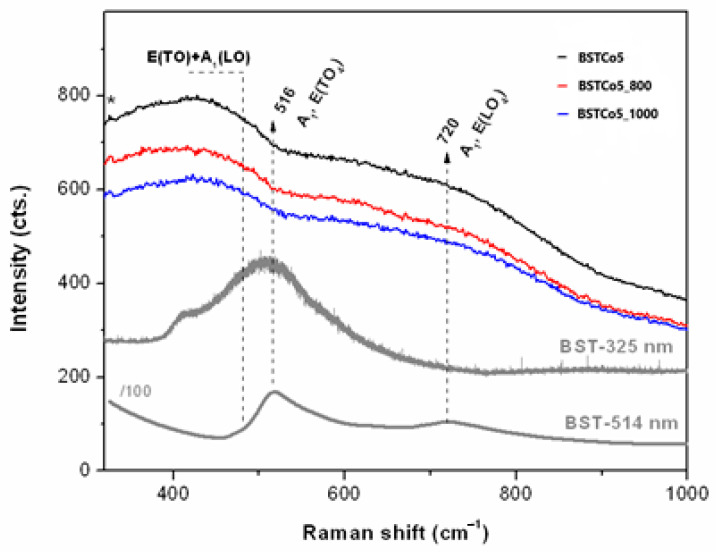
UV Raman spectra of the BST5Co samples compared with BST (*325 nm laser lines).

UV Raman spectroscopy (Figure 4) is a very sensitive technique for the surface investigation of materials, especially nanoscale ferroelectrics and their thin films [34].

UV Raman spectra of the Co-doped BaSrTiO_3_ were compared with those of pure BST in order to investigate the Co dopant influence on the BST structure, as illustrated in Figure 4. Both pure BST and BSTCo5 were synthesized under the same conditions.

Pure BST samples were excited with 325 and 514 nm laser lines. In both cases, the presence of cubic and tetragonal phases was observed. In the case of Co-doped BST samples, only the 325 nm laser source was used. The vibration corresponding to the tetragonal structure was not identified. In this case, it could be considered that the cubic phase was predominant.

Thus, given the forbidden Raman activity of the cubic ABO_3_ [36,37], the presence of a limited number of bands in Figure 4 indicated second-order Raman modes of the disordered cubic BaTiO_3_ (BT) in the as-obtained BST5Co5 sample. Wider bands were recorded for the BST5Co_800 sample. Typically, Raman spectrum of BT is divided into two ranges, below 250 cm^−1^ (cationic network and the lattice modes) and within the 200–800 cm^−1^ range, which is due to the bending and stretching modes of the oxygens in the covalent linked BO_6_ octahedra in ABO_3_ [38]. However, the edge filter prevented recording of the UV Raman spectra at low wavenumbers. Moreover, BT and SrTiO_3_ (ST) had several active modes close to each other [39]. The TO_4_ modes presented in the BST spectra in Figure 4 vanished for BST5Co samples where cubic structure prevailed. This was similar to the second-order spectral features reported for the paraelectric BT layers in the BaTiO_3_/SrTiO_3_ superlattices at temperatures exceeding Tc (>250 °C) [40]. Co-doping of the BST triggered the formation of the cubic phase.

The results obtained from the Raman analysis are in agreement with the results obtained by the XRD analysis.

### 3.4. Scanning Electron Microscopy for Co-Doped BaSrTiO_3_ Powder

**Figure 5:**
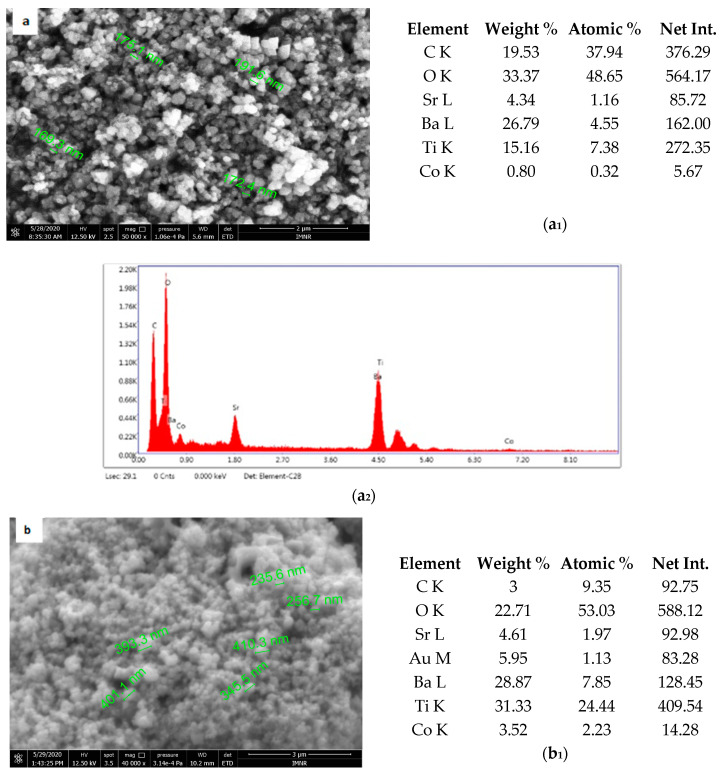

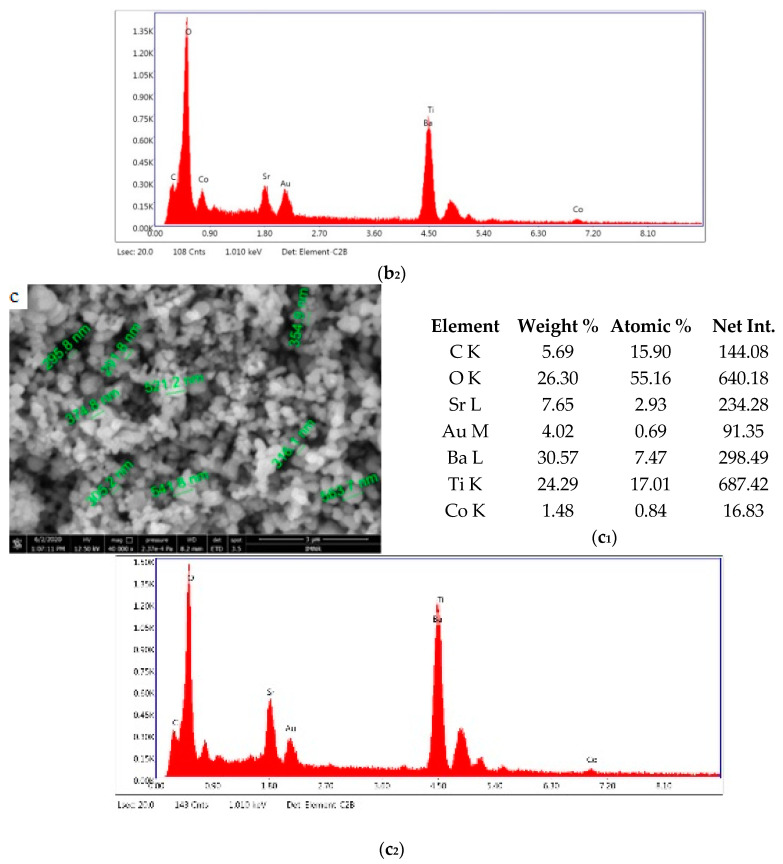
Sanning Electron Microscopy/EDAX analysis for BSTCo5, BSTCo5_800, and BSTCo5_1000: (**a**–**c**) Scanning Electron Microscopy for BSTCo5, BSTCo5_800, and BSTCo5_1000; (**a_1_**–**c_1_**) tables of EDAX elemental content for BSTCo5, BSTCo5_800, and BSTCo5_1000; (**a_2_**–**c_2_**) EDAX mapping for BSTCo5, BSTCo5_800, and BSTCo5_1000.

Figure 5 shows the results of morphological and structural analysis by Scanning Electron Microscopy. It can be seen that the BSTCo5 sample obtained in hydrothermal conditions contains aggregates of faceted particles with dimensions in the range of 170–190 nm. The morphology of the sample BSTCo5 is influenced by the heat treatment temperature (Figure 5b,c). In the sample that is heat-treated at 800 °C, particles have dimensions in the range 230–400 nm, and no important particle growth can be observed for the sample heat-treated at 1000 °C (290–560 nm). Thermal treatment temperature does not influence significantly the size of the particle.

The presence of two primary BST phases, cubic and tetragonal, identified by XRD analysis, likely hinders the particle growth [41]. The presence of Ba, Sr, Ti, and Co elements in each sample is shown in Figure 5. The EDAX analysis is in agreement with the XRD and chemical elemental analyses.

### 3.5. Dynamic Light Scattering Analysis for BSTCo5 Powder

The DLS analysis indicates the presence of nanosized particles by completing the information obtained by SEM analysis, providing essential data in the processing and determination of sensitive properties. Stable suspension based on BSTCo5 was prepared for the DLS measurements.

Hydrodynamic diameter of spherical particles can be defined using the Stokes–Einstein equation:

d_H_ = kT/3ηπ D (1)

where d_H_ = hydrodynamic diameter, k = Boltzmann’s constant (1.38 × 10^−23^ NmK^−1^), T = absolute temperature (K), η = solvent viscosity (N·s·m^−2^), and D = diffusion coefficient (m^2^·s^−1^).

**Figure 6:**
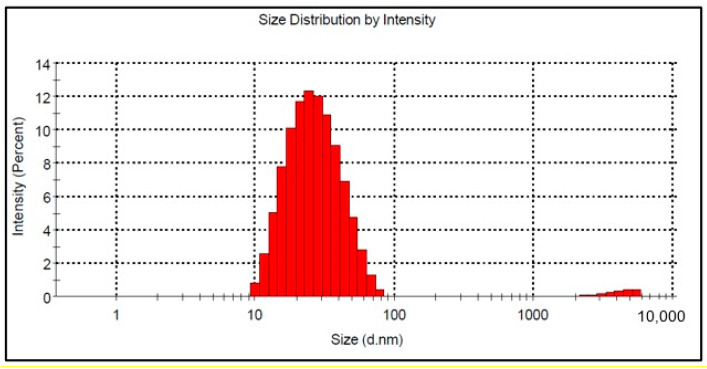
Mean size of BSTCo5 particles in suspension.

Figure 6 shows the particle size distribution by DLS analysis for the Co-doped BST sample. The particle size is in the range between 10 and 78 nm, and the calculated average hydrodynamic diameter of the nanoparticles is 24 nm (Figure 6). It is well known that nanoparticles based on BaTiO_3_ are prone to aggregation caused by interparticle interactions due to van der Waals forces [40].

By corroborating the XRD analysis for crystallite size with DLS analysis for BSTCo5, one can say that the sample is nanostructured, as presented by Simões et al. [41] for BST powder.

### 3.6. Differential Scanning Calorimetry and Thermogravimetry Analysis for BSTCo5 Powder

Hydrothermal obtained powders were used for RF sputtering target processing (pressing and sintering). The main reason these powders were subjected to thermal analysis was to determine the powder stability and phase transformations.

**Figure 7:**
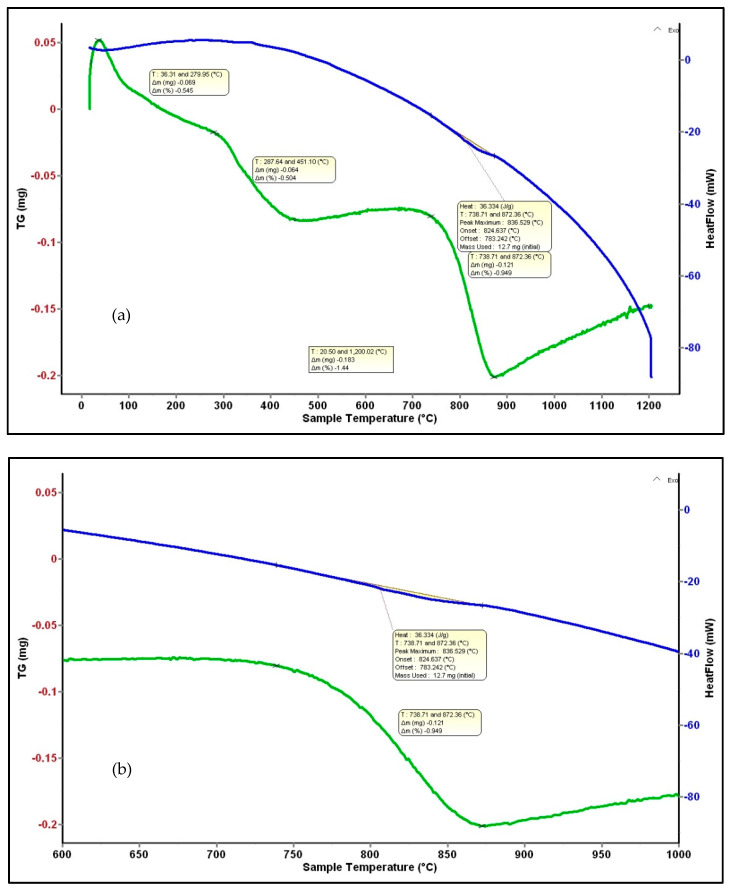
DSC-TG analysis of BSTCo5 powder (**a**) and a detail for the mass loss corresponding to the 836 °C peak (**b**).

The TG and DSC plots of the Co-doped BST powders heated from ambient temperature to 1200 °C are shown in Figure 7. Several mass losses are revealed in the TG curve, presented in Table 2. A first step is the release of adsorbed water in the range of 27–280 °C, the value of weight change being 0.6%. A second step, occurring from 280–450 °C, corresponds to a total weight loss of 0.5% and results from the release of chemisorbed water or hydroxyl groups. Between 450 and 730 °C, there is no mass loss, and between 730 and 870 °C, there is a mass loss of about 0.9% that is likely correlated with the polymorphic transformation of BaCO_3_ [42], and is also identified by XRD analysis. No weight loss is registered in the range of 870–1200 °C.

### 3.7. Thermal Conductivity Analysis for BSTCo5 Powder

The specific heat of the Co-doped BST material is used for estimating the thermal inertia of the sensitive layer of the sensor, with the aim of a quick sensor cleaning protocol. Thermal conduction in the sensing material also plays an important role in dissipating heat.

BSTCo5 samples treated at 800 °C were subjected to thermal conductivity, thermal diffusivity, and volumetric specific heat analysis (Table 3). Based on the volumetric specific heat, the specific heat of the BSTCo5 sample was calculated. Ten successive measurements were performed in order to obtain the values in Table 3.

The thermal conductivity (room temperature) of the BSTCo5 sample is 0.4 W/(mK). Compared with a similar chemical compound, BaTiO_3_, this is smaller than the values of 2.61 to 2.85 W/(mK) reported by others some decades ago [43], but it is consistent with recent data presented by Shirane [44]. The value of 0.576 (J/gK) for specific heat is in good agreement with the literature reports of Popescu et al. [45] and Shirane et al. [44], and it is consistent with literature data of pure BaTiO_3_ [43,44,45,46]. Accordingly, the 0.26 (mm^2^/s) value for thermal diffusivity is lower than 1.03 to 1.18 (mm^2^/s), reported by He [42] for BaTiO_3_ samples. BT sample A represents BaTiO_3_ (Supplier A), and BT sample B represents BaTiO_3_ (Supplier B) [43].

The variation of the data can be attributed to: (a) dopants used for BaTiO_3_ based material, (b) different manufacturing procedures leading to lower densities of the material and, to some degree, (c) techniques used for thermal conductivity measurement. To be more specific, a drop in thermal conductivity for Co-doped BST compared with BT can be explained by the influences of the dopant, which induce defects in the crystal structure.

### 3.8. CO_2_ Sensing Properties

**Figure 8:**
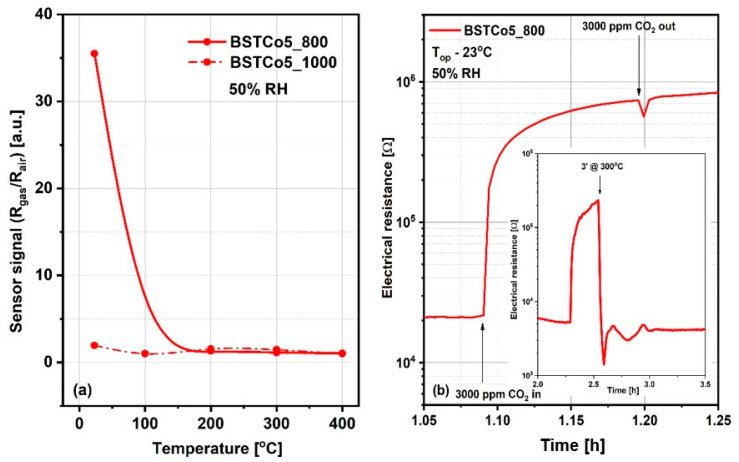
(**a**) Sensor signal for 3000 ppm CO_2_ for temperature range 23–400 °C; (**b**) Surface contamination from exposure to CO_2_ and the solution of 3 minutes at 300 °C temperature boost applied for its rapid cleaning (b-inset).

It is generally accepted knowledge that, for chemo-resistive sensors, the chemical interactions of gases are modulated by the temperature of the sensitive layers and determine the variation of their electrical resistance. Thus, the preliminary selection between BSTCo5_800 and BSTCo5_1000 sensors was assessed for a fixed CO_2_ concentration of 3000 ppm in the temperature range of room temperature (RT) between 23 and 400 °C. CO_2_ was dosed into synthetic airflow with 50% RH, an atmosphere similar to in-field background (real operating conditions). Accordingly, the sensor signal, defined as the relative measure of the sensitive layer resistance under CO_2_ exposure (R_gas_) with respect to the reference atmosphere (R_air_), is represented in Figure 8a. As can be seen, the highest signal is obtained for the BSTCo5_800 layer at room temperature (RT = 23 °C). A possible explanation is related to the specific structure and morphology of the samples inducing specific surface defects [46]. Despite the magnitude of the signal greater than 30 and the good response time, the electrical resistance of the BSTCo5_800 layer does not return to its initial value after exposure to CO_2_, most likely due to the formation of irreversible carbonic acid at the surface (Figure 8b). A short temperature boost of 3 min (‘) at 300 °C ensures the cleaning of the surface and the return of the resistance, as seen in the inset from Figure 8b.

Knowing that CO_2_ solubility in water at room temperature is around 1.25 g/kg H_2_O, the following relation may take place:

(2) $$CO_{2}{}^{gas}+H_{2}O↔H_{2}CHO_{3}↔H^{+}+HCO_{3}^{-}$$

**Figure 9:**
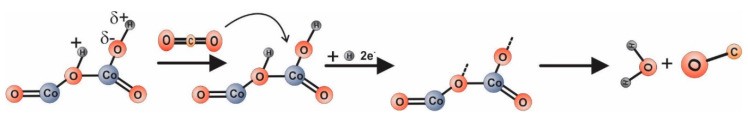
Possible conduction mechanism involved in CO_2_ detection

Alternatively, (see Figure 9) CO_2_ reacts with hydrogen from the dissociated water. We note that the electric field specific to the electrical measurements is 15 kV/m.

(3) $$CO_{2}{}^{gas}+2e^{-}+2H^{+}\to CO+H_{2}O$$

For the case of Equation (2), the changes in electrical resistance are induced by water-mediated dipole interactions resulting from physisorbed water vapors from the atmosphere [44]. Such behavior is in good correlation with the water condensation phenomena triggered by the porous structure as revealed from the TG and DSC plots.

Knowing that nanoparticles based on BaTiO_3_ are prone to aggregation, which is caused by inter-particle interactions due to van der Waals forces, capillary condensation of the water vapors occurs at the liquid-vapor equilibrium with respect to the Kelvin equation:

(4) $$r=\frac{-2\gamma V_{L}cos\theta }{RTln\left(p/p_{0}\right)}$$

where θ is the contact angle between the liquid and the wall of the capillary pore, V_L_ represents the molecular volume of liquid, γ represents the water’s surface tension, R represents a gas specific constant, T represents the absolute temperature express in K, and r is the Kelvin radius of the capillary pores.

The case in Equation (3) explains the increase of resistance by electrons involved in CO_2_ decomposition in the presence of dissociated water (due to the high intensity of the electric field) [47].

**Figure 10:**
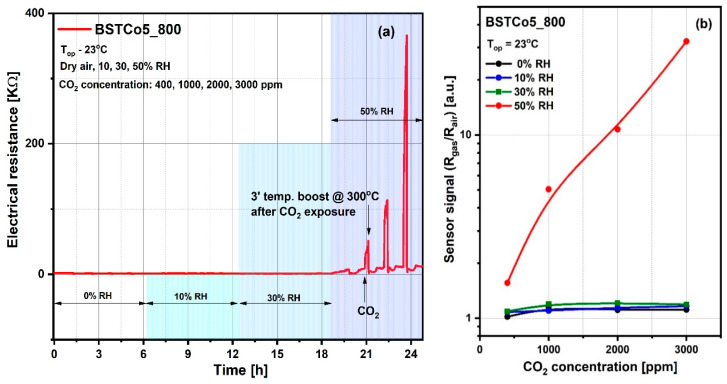
(**a**) Variations in electrical resistance for BSTCo5_800 due to exposure to CO_2_ in atmospheres with variable relative humidity (0%, 10%, 30%, and 50% RH); (**b**) The sensor signal for 400–3000 ppm of CO_2_ under different relative humidity background conditions.

Accordingly, the influence of the relative humidity level on the reference atmosphere (Rair) as well as on the sensitive layer resistance under CO_2_ exposure (Rgas) have been highlighted through electrical investigations, and prove that the value of 50% RH (known as medium level value for in-field atmosphere) constitutes the threshold of the CO_2_ detection regime (Figure 10). As can be seen in Figure 10a, the CO_2_ interaction is favored by the relative humidity, the threshold being 50% RH. The stability of the electrical resistance up to this threshold suggests the validity of the dipole interaction without the change of electric charge, described by Equation (2). Above this threshold, there is an increase in resistance following the exposure to progressive concentrations of CO_2_, which demonstrates a decrease in the number of charge carriers, suggesting the validity of Equation (3). We have to mention that after each CO_2_ concentration, a short temperature boost of 3′ at 300 °C was applied for the cleaning of the surface and the return of the resistance, as explained for the Figure 8b. For the simplicity of Figure 10a, the moment of CO_2_ admission and the moment of the temperature boost is shown with arrows only for one of the four CO_2_ concentrations (400, 1000, 2000, and 3000 ppm). The response and recovery transients have also been calculated (see Table 4).

The response time refers to the time needed to reach a stable sensor response after a stepwise increase in the test gas concentration. Hence, the response time is defined as the time τ_response_ takes for 90% of the sensor’s electrical resistance to be accomplished.

The recovery time refers to the time the sensor needs for the sensor response to return to the base electrical resistance after removing the test gas. The recovery time is defined in a similar manner as the response time, namely τ_recovery_ takes 90% of the sensor’s electrical resistance to return to its base resistance.

The sensor signal (Figure 10b) is defined as the ratio between the electrical resistance under CO_2_ exposure (R_gas_) and the electrical resistance under synthetic air with 50% RH (R_air_).

The sensor signal is represented for the range of CO_2_ concentrations between 400 and 3000 ppm. The lower concentration of 400 ppm corresponds to the current safe level of CO_2_ for outdoor ambient air. Typical safe concentrations for indoor spaces are 400–1000 ppm CO_2_, with upper levels between 1000 and 5000 ppm CO_2_ causing headaches, sleepiness, loss of attention, increased heart rate, and slight nausea. Accordingly, to prevent the health risks associated with CO_2_, the imposed exposure limit is 5000 ppm [48].

**Figure 11:**
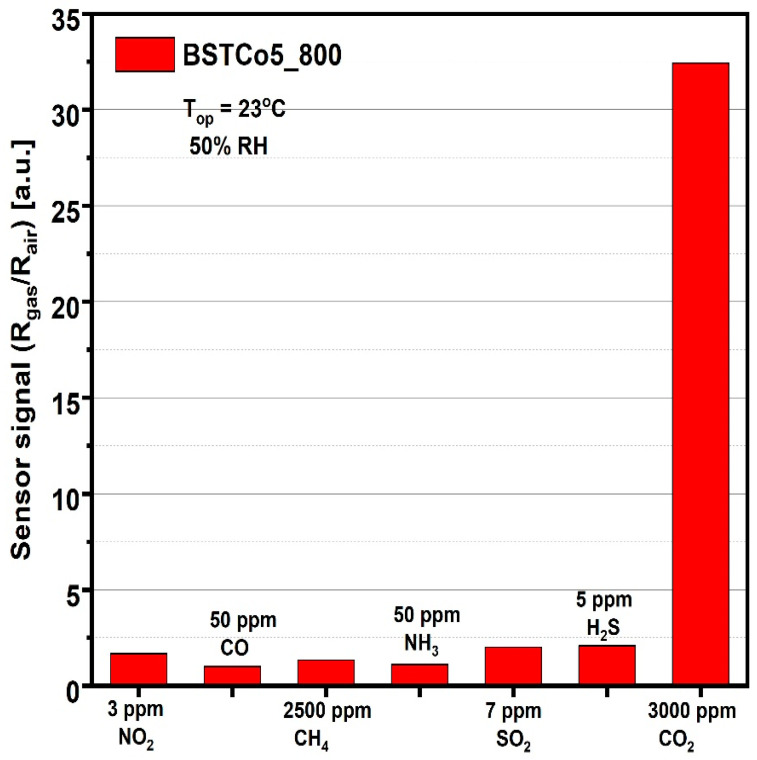
Sensor signal for 3000 ppm CO_2_ and for other potential interfering gases.

The last step in evaluating the sensitive properties of BSTCo5_800 was the selectivity analysis that highlighted the signal to CO_2_ compared with the signals to other gases potentially present in the ambient atmosphere. For each of the gases, the test was performed at the specific detection threshold stipulated by European regulations (Figure 11).

Considering the simple and low-cost technology, the good signal under infield humidity conditions (Figure 10a), the high selectivity relative to other potential interfering gases (Figure 11), and the low power consumption corresponding to RT operation, we can say that BSTCo5_800 has the potential for future portable detector development.

For other potential interfering gases, the concentrations were chosen according to the EU detection limits [25].

## 4. Conclusions

In the present paper, chemical, microstructural, physical, and thermodynamic properties of BSTCo5 powder obtained in hydrothermal conditions were investigated. For a proper evaluation of potential applications as a sensing material in CO_2_ detection, the composition and thermodynamic stability of the powder was first assessed at 800 and 1000 °C.

It was shown that BSTCo5 was stable at 1200 °C, according to DSC/TG analysis. BSTCo5, as an obtained nanopowder, consisted of a mixture of phases (cubic and tetragonal) at 1000 °C. It was likely that its cubic structure prevailed, which was also supported by Raman spectroscopy.

Thermal conductivity, thermal diffusivity, and specific heat were consistent with literature data for BaTiO_3_ compounds, and the variation of data occurred due to the dopants used, as well as the different manufacturing procedures and different techniques used for thermal conductivity measurement. Further studies may aim at improving thermal conductivity properties using co-doping with mixed rare-earth oxides.

The BSTCo5 that was heat-treated at 800 °C, encoded BSTCo5_800, presented high selectivity for CO_2_ related to other potential interfering gases. This ability, together with the good signal, simple and low-cost technology, and the low power consumption corresponding to RT operation, suggested that BSTCo5_800 had good development potential for portable CO_2_ detectors. Two possible mechanisms of carbon dioxide (CO_2_) detection via electrical resistance modification were also proposed under real operating conditions.

## Figures and Tables

**Table 1:** The results of elemental analysis determined by ICP-OES.

Chemical Analysis wt.%			
Ba	Sr	Ti	Co
41.2 ± 0.5	10.2 ± 0.7	21.1 ± 0.4	1.35 ± 0.3

**Table 2:** Heat flow and mass loss for BSTCo5.

Temperature (°C)	ΔH (J/mol)	Mass Loss (mg)	Mass Loss (%)	Observation
27–280	-	0.069	0.6	Release of Adsorbed Water
280–450	-	0.064	0.5	Chemisorbed Water or Hydroxyl Groups
450–730	-	-	-	No Mass Loss
836	8038	0.121	0.1	Polymorphic Transformation of BaCO_3_

**Table 3:** Thermal conductivity, thermal diffusivity, volumetric specific heat, and specific heat determined at room temperature.

Material	k (W/mK)	α (mm^2^/s)	Volumetric Cp (10^6^ J/m^3^K)	Cp (J/gK)	Ref.
BSTCo5	0.40 ± 0.01	0.26 ± 0.02	1.513 ± 0.09	0.576	Present work
BT Provider A	2.61 ± 0.02	1.03 ± 0.01	2.532 calculated	0.434 ± 0.004	[42]
BT Provider B	2.85 ± 0.04	1.18 ± 0.02	2.411 calculated	0.406 ± 0.008	

**Table 4:** Calculated response and recovery times related to the changes in the electrical resistance upon different CO_2_ concentration exposure as presented in Figure 10a.

50% RH	400 ppm CO_2_		1000 ppm CO_2_		2000 ppm CO_2_		3000 ppm CO_2_	
Transients	τ_response_	τ_recovery_	τ_response_	τ_recovery_	τ_response_	τ_recovery_	τ_response_	τ_recovery_
Minutes	9	8	13	7	6	7	12	8
